# miR-1204 promotes hepatocellular carcinoma progression through activating MAPK and c-Jun/AP1 signaling by targeting ZNF418 : Retraction

**DOI:** 10.7150/ijbs.75583

**Published:** 2022-06-07

**Authors:** Liang Wang, Liankang Sun, Yufeng Wang, Bowen Yao, Runkun Liu, Tianxiang Chen, Kangsheng Tu, Qingguang Liu, Zhikui Liu

**Affiliations:** Department of Hepatobiliary Surgery, the First Affiliated Hospital of Xi'an Jiaotong University, Xi'an, Shaanxi, China, 710061.

This retracts the article “miR-1204 promotes hepatocellular carcinoma progression through activating MAPK and c-Jun/AP1 signaling by targeting ZNF418” in *Int J Biol Sci* 2019; 15(7): 1514-1522. doi: 10.7150/ijbs.33658.

The article (miR-1204 promotes hepatocellular carcinoma progression through activating MAPK and c-Jun/AP1 signaling by targeting ZNF418) has been retracted since the authors noticed that there were some unreliable data in the experimental validations. After careful consideration, the authors decided to retract this article. We truly regret any inconvenience caused by our careless and do apologize for any unintentional mistakes.

All authors have agreed to the retraction of the article.

